# Child With Extranodal Marginal Zone B-cell Lymphoma of Mucosa-associated Lymphoid Tissue

**DOI:** 10.1097/PG9.0000000000000120

**Published:** 2021-08-26

**Authors:** Khalid A. Alghamdi, Alaa M. Bokhari, Imad A. El Hag

**Affiliations:** From the * Pediatric Department, Security Forces Hospital, Riyadh, Saudi Arabia; † Histopathology Department, Security Forces Hospital, Riyadh, Saudi Arabia.

**Keywords:** Mucosa-associated lymphoid tissue lymphoma, MALToma, Helicobacter pylori

## Abstract

Many patients present to our clinic with *Helicobacter pylori* (*H. pylori*) infection. Most have nonspecific symptoms that cannot be immediately attributed to *H. pylori*. The joint European Society for Paediatric Gastroenterology, Hepatology, and Nutrition (ESPGHAN)/North American Society for Pediatric Gastroenterology, Hepatology, and Nutrition (NASPGHAN) guidelines recommend upper gastroscopy to detect the cause of such symptoms. Herein, we present the case of a 9-year-old girl diagnosed with gastric mucosa-associated lymphoid tissue lymphoma associated with *H. pylori* infection using upper gastroscopy. We believe that a patient of such a young age with this serious condition secondary to *H. pylori* will highlight the importance of upper gastrointestinal endoscopy in such cases.

## INTRODUCTION

Extranodal marginal zone B-cell lymphoma (EMZBL) of the mucosa-associated lymphoid tissue (MALT) is very rare in children (1). For EMZBL to occur, underlying chronic inflammation of the stomach needs to develop and subsequently transform into lymphoma, a process that usually takes decades, and this is very unlikely in young children.

*Helicobacter pylori* (*H. pylori*) is the main organism involved in chronic inflammation of the stomach, leading to gastric MALT lymphoma (2) as well as being one of the most common bacteria of chronic infections. Approximately half of the world’s population is infected; however, the distribution is not homogeneous. It is the main etiological factor for gastritis and peptic ulcers (3) and is consistently recognized as the leading etiologic agent of gastric cancer. Less than 0.5% of patients with *H. pylori* develop MALT lymphoma (4).

In children, *H. pylori* prevalence varies. It is seen more frequently in developing countries (up to 55%) and increases in prevalence with age. However, recent studies have revealed a decreased prevalence worldwide. Transmission data support both intrafamilial transmission, especially from mother to child, as well as extrafamilial transmission (5).

## CASE REPORT

A previously healthy 9-year-old girl presented to the outpatient clinic of the Security Forces Hospital with a 3-month history of increasingly severe, persistent abdominal pain, that was mainly epigastric. Other symptoms included nausea, weight loss (3 kg), and a decrease in appetite and energy level. Bowel movements were irregular during this period. One week before the presentation, the patient’s stool became black-colored. Dark blood and clots were noted in the stool at 2 instances. There was no history of unexplained fever or night sweating.

**FIGURE 1.**
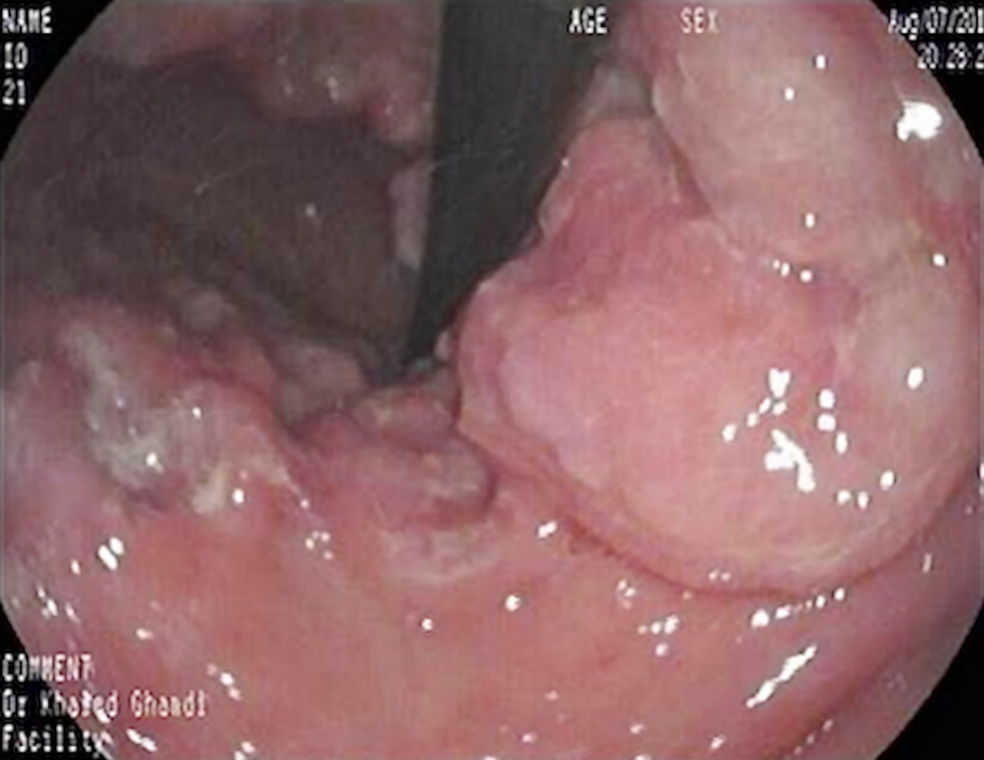
Large, fungating lesion with irregular lobulation seen in the stomach.

**FIGURE 2.**
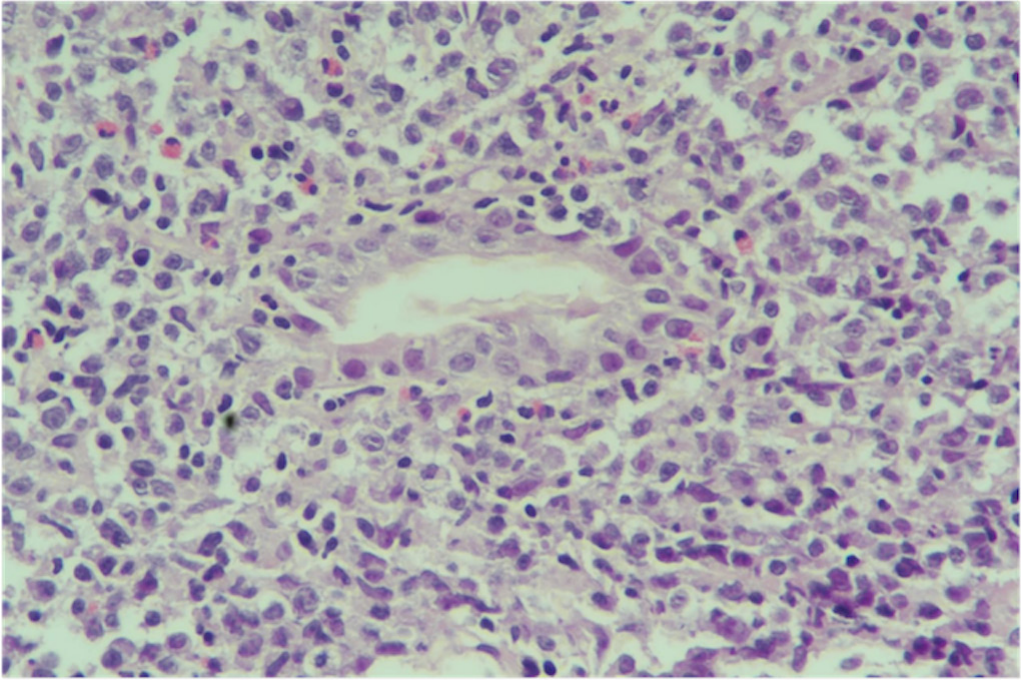
Diffusely infiltrated gastric mucosa by atypical small to medium size lymphoid cells with a lymphoepithelial lesion, hematoxylin and eosin stain.

At the time of presentation, the patient weighed 19.5 kg and looked ill, thin, and pale. The abdomen was soft, with significant tenderness over the epigastric area. The rest of the examination was unremarkable. Laboratory values are presented in Table 1. Gastroduodenoscopy revealed an ulcerated gastric mass with an irregular surface, occupying most of the fundus and body (Fig. 1). Significant mucosal nodularity was observed in the antrum. The *H. pylori* urease test was positive. Multiple biopsies were sampled from the lesion and other parts of the stomach, as recommended with any gastric lesion (based on gastric mapping). These biopsies revealed a lymphoepithelial lesion (Fig. 2). Immunohistochemical stains CD20 and CD43 were positive, while CD10 and CD5 immunostaining were negative. Active chronic *H. pylori* gastritis was evident from antral biopsies.

**TABLE 1.** Laboratory test results

Test	Result	Normal range
White blood cells	10.49 × 10^9^/L	4.5–13.5 × 10^9^/L
Hemoglobin	72 g/L	110–160 g/L
MCV	63 fL	77–98 fL
Platelets	684 × 10^9^/L	150–400 × 10^9^/L
C-reactive protein	183 mg/L	<5 mg/L
ESR	43 mm/hour	0–20 mm/hour
Albumin	24 g/L	38–40 g/L
LDH	254 g/L	120–300 g/L
Occult blood	Positive	Negative
Stool for *Helicobacter pylori*	Positive	Negative

ESR = erythrocyte sedimentation rate; LDH = lactate dehydrogenase; MCV = main corpuscular volume

Chest, abdomen, and pelvic computed tomography scans revealed extensive diffuse gastric wall thickening, extending along the fundus, body, and antrum region, with multiple enlarged mesenteric lymph nodes at the lesser sac only.

The patient received *H. pylori* eradication therapy (amoxicillin, clarithromycin, and proton pump inhibitor) for 2 weeks. One week after completing the course, a gastroscopy was repeated and did not show any change in the mass. Furthermore, *H. pylori* were not detected in the biopsies.

Due to the advanced stage of the patient, she was referred to the oncology center for further management. She received 6 cycles of chemotherapy (rituximab plus cyclophosphamide, doxorubicin, vincristine, and prednisone), which lasted for 6 months.

After completing the chemotherapy, she underwent gastroscopy with multiple gastric biopsies, which revealed complete remission. Three months later, gastroscopy was repeated and biopsies were taken, with no signs of relapse. Gastroscopy with gastric biopsies was performed every 6 months for the last 2 years with no signs of recurrence.

## DISCUSSION

The most frequent site of extranodal Non-Hodgkin’s lymphoma is the gastrointestinal tract. More than 70% of primary gastrointestinal lymphomas present as gastric lymphoma. Marginal zone B-cell lymphomas of MALT are predominant. The second most common type of gastric lymphoma is diffuse large B-cell lymphoma. T-cell lymphomas are extremely rare in the stomach (6).

This case report describes a case of gastric MALT lymphoma in a 9-year-old girl. To the authors’ knowledge, she is the youngest reported patient with gastric MALT lymphoma. *H. pylori*, recognized as the leading etiologic agent of gastric cancer, plays an important role in the development of almost all gastric MALT lymphomas (2).

Gastric tissue normally does not contain MALT but may acquire it in a secondary process with background chronic antigenic stimulation such as *H. pylori* (6). For this reason, it is highly unlikely in very young patients.

The risk of gastric MALT lymphoma secondary to *H. pylori* infection is very rare but should be kept in mind. Only 3% of infected patients develop gastric adenocarcinoma, with less than 0.5 % developing MALT lymphoma (4). Its clinical course is generally indolent, and most patients are diagnosed with early-stage I or II of the disease (1). The patient, in this case report, presented with significant clinical symptoms and advanced stage gastric MALT lymphoma from the beginning. The prognosis of pediatric EMZBL is reported to be excellent (7), as seen in this patient, despite the advanced stage. She responded very well to *H. pylori* eradication and chemotherapy.

Eradication therapy for *H. pylori* should be based on culture and strain susceptibility as recommended by the NASPGHAN/ESPGHAN guidelines, but this was not available in our laboratory. Therefore, empirically she was given the clarithromycin-based regimen. However, in many countries due to increasing clarithromycin resistance, metronidazole-based regimen consider first-line therapy. The ESPGHAN/NASPGHAN guidelines recommend that the effectiveness of first-line therapy be evaluated in national/regional centers.

To diagnose MALT lymphoma, a sufficient number of biopsies from macroscopic lesions and normal-appearing mucosa (based on gastric mapping) are essential (6). Once the diagnosis is established, a staging procedure follows. Screening chest, abdomen, and pelvic computed tomography scans are carried out. Endoscopic ultrasound and bone marrow biopsy are strongly recommended. Endoscopic ultrasound can determine the depth of infiltration and detect the presence of enlarged epigastric lymph nodes (2). Accurate diagnosis and staging are important before starting treatment.

All patients with gastric MALT lymphoma should receive *H. pylori* eradication therapy, regardless of disease stage. In patients with stage I/II, complete remission is achieved in 50%–90% of cases with *H. pylori* eradication alone (8). Patients who do not respond to *H. pylori* eradication alone or who have advance stage III or IV need further oncology treatment such as radiotherapy or chemotherapy (8).
